# The War and Infant Welfare

**Published:** 1915-09-25

**Authors:** 


### THE WAR AND INFANT WELFARE.

At the meeting of the Royal Sanitary Institute held at Brighton on the 4th inst., the question of child welfare and the conservation of infant life at this critical period in the history of the nation came up for thoughtful and sustained discussion. The drain on the virile manhood of the nation due to the ravages of war, the high infant death-rate in certain districts, and the decreasing birth-rate give cause for no little anxiety with regard to the future. While it is true that for years there has been a steady decrease in the death-rate, this fall cannot continue indefinitely and in war-time will receive a sudden check. Moreover, from the view-point of national welfare the fall in the death-rate, which has been the feature of the last few decades, has now reached a stage at which it is of much less significance, the reasons for this being that a further appreciable fall will require a greatly increased effort, and that *pari passu* with the decreasing death-rate during recent years there has been a striking fall in the birth-rate. The seriousness of this problem was fully reflected in the papers read at Brighton. Dr. Boobbyer, medical officer of health for Nottingham, discussed the high infant death-rate in urban districts compared with rural districts, and pointed out the serious significance of a high urban infant death-rate in view of the tendency of the rural population to flock to the towns. He emphasised the suicidal folly of the present-day limitation of families and of the importance to the Empire of having the stock properly propagated and nurtured. Dr. Forbes, medical officer of health for Brighton, dealt with infant mortality and the birth-rate, and pointed out that increase in the latter was of greater national importance than a decrease in the infant death-rate. He advocated substantial modifications of taxes in the case of those with large families, and legislative measures to encourage an increased birth-rate. Other speakers voiced somewhat similar views with regard to State assistance to parents with large families. In reviewing this important question one is forced to the conclusion that the kernel of the whole problem is to be found in the trend of modern civilisation towards the voluntary restriction of families. The causes underlying this national evil are no doubt partly economic, but it is also recognised that money which was expended by our parents in the rearing of children is to-day spent in great measure on sport and pleasure. It is not as yet possible to estimate to what extent the war may alter this or to gauge how far the women of England realise their responsibility, but the problem is one which at the present time of crisis calls for grave consideration.

